# Co-Crystal Formation of Antibiotic Nitrofurantoin Drug and Melamine Co-Former Based on a Vibrational Spectroscopic Study

**DOI:** 10.3390/pharmaceutics11020056

**Published:** 2019-01-30

**Authors:** Ziming Zhang, Qiang Cai, Jiadan Xue, Jianyuan Qin, Jianjun Liu, Yong Du

**Affiliations:** 1 Centre for THz Research, China Jiliang University, Hangzhou 310018, China; ziming_zhang@yeah.net (Z.Z.); northwestun@163.com (Q.C.); jyqin@cjlu.edu.cn (J.Q.); jianjun@cjlu.edu.cn (J.L.); 2 Department of Chemistry, Zhejiang Sci-Tech University, Hangzhou 310018, China; jenniexue@126.com

**Keywords:** nitrofurantoin (NF), melamine (MELA), hydrated co-crystal, terahertz time-domain spectroscopy (THz-TDS), Raman spectroscopy, density functional theory (DFT)

## Abstract

The co-crystallization of active pharmaceutical ingredients (APIs) has received increasing attention due to the modulation of the relative physicochemical properties of APIs such as low solubility, weak permeability and relatively inferior oral bioavailability. Crystal engineering plays a decisive role in the systematic design and synthesis of co-crystals by means of exerting control on the inter-molecular interactions. The characterization and detection of such co-crystal formations plays an essential role in the field of pharmaceutical research and development. In this work, nitrofurantoin (NF), melamine (MELA) and their hydrated co-crystal form were characterized and analyzed by using terahertz time-domain spectroscopy (THz-TDS) and Raman vibrational spectroscopy. According to the experimental THz spectra, the hydrated co-crystal form has characteristic absorption peaks at 0.67, 1.05, 1.50 and 1.73 THz, while the THz spectra for the two raw parent materials (NF and MELA) are quite different within this spectral region. Similar observations were made from the experimental Raman vibrational spectra results. Density functional theory (DFT) calculation was performed to help determine the major vibrational modes of the hydrated co-crystal between nitrofurantoin and melamine, as well as identify the structural changes due to inter- and/or intra-molecular hydrogen bonding motifs between NF and MELA. The results of the theoretical frequency calculations corroborate the THz and Raman experimental spectra. The characteristic bands of the NF–MELA-hydrated co-crystal between nitrofurantoin and melamine were also determined based on the DFT simulated calculation. The reported results in this work provide us with a wealth of structural information and a unique vibrational spectroscopic method for characterizing the composition of specific co-crystals and inter-molecular hydrogen bonding interactions upon pharmaceutical co-crystallization.

## 1. Introduction

**Figure 1:**
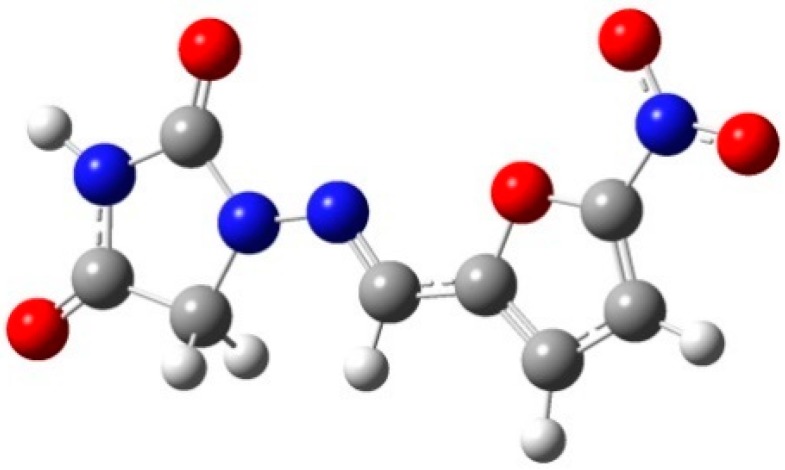
Molecular structure of nitrofurantoin (NF).

Nitrofurantoin (NF, with its molecular structure shown in Figure 1) is a widely used antibacterial drug for the oral treatment of urinary tract infections [1,2]. This drug is on the World Health Organization Model List of essential medicines and belongs to the Class IV drugs according to the Biopharmaceutics Classification System (BCS), that is, it shows low solubility, weak permeability and relatively inferior bioavailability [3]. It has been reported that the dissolution rate and bioavailability of NF decreases gradually upon storage under different temperature and relative humidity conditions [4,5]. On the other hand, NF is known to be a photosensitive drug [6,7,8], and exposure to natural or fluorescent light has been reported to cause discoloration in the NF crystals and solutions [9]. These characteristics can significantly affect the physiochemical properties of the active pharmaceutical ingredients (APIs) in NF. Hence, it is important to explore various solid forms of NF that could enhance its solubility, dissolution profile and bioavailability.

The co-crystallization and salt formation of APIs are common methods to modulate relative physicochemical properties such as solubility, stability or bioavailability [10,11,12]. In fact, the salt forms have some limitations compared with co-crystals. Salt formation is confined to acid–base reactions, governed by an appropriate ΔpK_a_ value, while co-crystallization offers a different pathway, where any pharmaceutical substance could potentially be co-crystallized without having ionizable groups [13,14]. Furthermore, there is a great number of potential non-toxic co-crystal formers (CCFs) that could be used in co-crystal formations [15]. The most important advantage of the co-crystals is that the changes occurring in the crystal packing of the specific drug could lead to improved variation of the corresponding API’s physicochemical properties, whereas the active ingredient and therapeutic effect of the drug are generally not influenced [16].

Crystal engineering plays a decisive role in the systematic design and synthesis of co-crystals by means of exerting some control on the specific inter-molecular interactions [17,18,19]. Usually, the hydrogen bond is the most common interaction, and co-crystal formation can be rationalized considering the presence of both hydrogen bond donors and acceptors in the starting parent molecules. Ashwini Nangia et al. [20] prepared the NF-4-aminobenzoic acid co-crystal and NF-urea co-crystal successfully, and then powder X-ray diffraction (PXRD), single-crystal X-ray diffraction (SC-XRD), differential scanning calorimetry (DSC) and thermogravimetric analysis (TGA) relative technologies were used to study the above mentioned co-crystals. They found that all the co-crystal forms significantly improved the corresponding intrinsic dissolution rates and the stability. Venu R. Vangala et al. [21] reported several co-crystals of NF with eight different kinds of CCFs such as 3-aminobenzoic acid, 4-aminobenzoic acid, urea, phenazine, melamine, etc. The physicochemical properties of these co-crystals, such as the aqueous solubility and photo-stability, were enhanced significantly and their results suggested that co-crystallization could be a viable alternative for improving the biopharmaceutical properties of APIs. Du et al. [22] investigated the structure and dynamic formation process of the NF-4-aminobenzoic acid co-crystal with typical vibrational spectroscopy, and the results indicated that the spectroscopic techniques could offer an attractive experimental method to identify and characterize pharmaceutical co-crystal formation and its dynamic process. Vibrational spectroscopic techniques include mid-infrared, Raman and terahertz (THz) spectroscopy, which have already been successfully used to explain co-crystallization of different solid-state pharmaceuticals [23,24,25,26].

**Figure 2:**
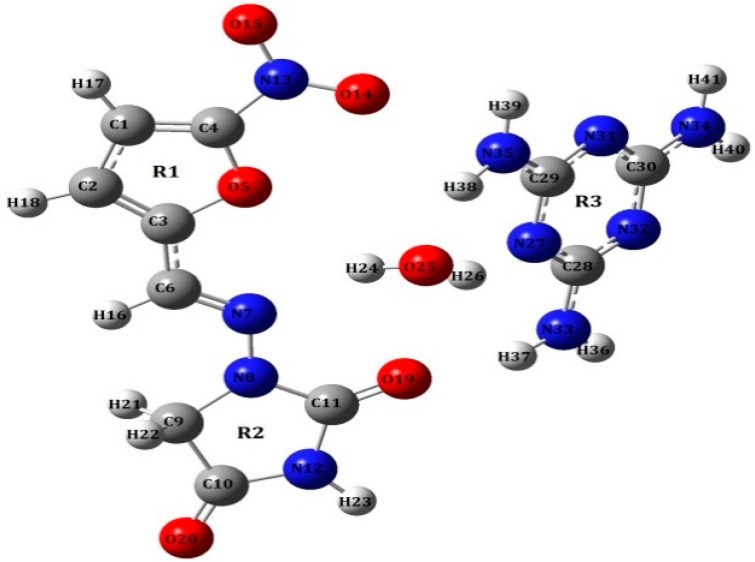
Molecular structure of the hydrated co-crystal formed between NF and melamine (MELA) reproduced from a previous reference [21].

In this work, the Raman and THz vibrational spectroscopy of a typical co-crystal system formed between NF and the co-crystal co-former melamine (MELA) was performed—under the solvent slow-evaporation method which was reported by Venu R. Vangala et al. [21]—and its molecular structure, with water strongly bonding to the azine N of NF and the pyridyl N of MELA based on Vangala’s previous work [21], is shown in Figure 2. The significant differences in the vibrational THz and Raman spectra of such hydrated co-crystals compared with the starting parent compounds could be observed. The density functional theory (DFT) was used to simulate the optimized structure and the vibrational modes of the theoretical hydrated co-crystal formed between NF, MELA and the water molecule at the microscopic molecular level. The theoretical calculations and experimental results were compared to help analyze the structure and the vibrational modes of the NF–MELA-hydrated co-crystal due to the inter-molecular hydrogen bonding effects between NF and MELA.

## 2. Materials and Methods 

### 2.1. Chemicals and Sample Preparation

The active pharmaceutical ingredient, NF, and the corresponding co-former, MELA, were both directly purchased from the Sigma-Aldrich Company (Shanghai, China) and were used without further purification. The solvents were of analytical grade. NF and MELA samples were ground before mixing to achieve particles with the mean size of several micrometers in order to minimize the scattering effects from the sample particles in the following THz spectroscopic measurements.

NF and MELA were mixed in the stoichiometric ratio of 1:1 and then dissolved in the hot ethanol–water solution, the volume of which was in the ratio 3:1, at a temperature of approximately 70 ℃. The solution was maintained in an ambient environment to allow slow evaporation and the solid state of the resulting NF–MELA-hydrated co-crystal form appeared as yellow blocks after several days.

### 2.2. THz and Raman Spectroscopic Characterization

The Z2 measurement system (Zomega Co. Ltd., New York, NY, USA) was adopted in THz-TDS measurements with a GaAs photoconductive antenna as the THz emitter and a ZnTe electro-optical crystal as the THz detector. A Femtosecond pulse laser (Spectra Physics, Owen, CA, USA) was used as the excitation light source with the repetition frequency set to 80 MHz, a pulse width of 100 fs and a center wavelength of 780 nm. The samples were poured into a steel die and subjected to ~4 MPa pressure for around 2 min. The resulting 13 mm in diameter, 1.5 mm-thick sample discs were obtained and sealed in plastic bags before further THz spectral measurement and analysis. All samples were tested at room temperature and the relative humidity of the sample cavity was kept in less than 0.1% by purging nitrogen gas during measurements at ambient conditions. A total of three THz spectra, representing three complete sets of sample and reference measurements, were averaged for each final THz spectrum. The THz electric field with time-domain was recorded for the reference (without sample holder) and each sample, and then, after the fast Fourier transform operation, the THz absorption spectrum was obtained by dividing the sample frequency response by that of the reference.

The Raman spectra were recorded at room temperature by using the Fourier transform Raman spectrometer (Thermo Nicolet Corporation, Madison, WI, USA). As for Raman spectral measurements, there was no need for further sample preparation. The spectra were acquired over 256 scans with a 2 cm^−1^ spectral resolution, over the frequency range 200–3500 cm^−1^, with a near-infrared laser operating power ~150 mW. The total analysis time per sample was of approximately 5 min intervals.

### 2.3. Theorectical Calculations

Quantum chemistry theoretical calculations were used to simulate the structures of the NF single molecule, MELA single molecule and their hydrated co-crystal NF–MELA form. All calculations were performed using the B3LYP functional [27,28,29]. The reliability of the B3LYP functional in calculations of ground-state geometries has been widely assessed previously [30]. The optimized geometries and corresponding vibrational frequencies of NF, MELA and its hydrated co-crystal form were computed within DFT by using the Gaussian 03 program [31] as the basis set of 6-311G(d,p). As for the simulated spectra, lorentzian line shapes were convolved into the calculated modes with a full-width half-maximum (FWHM) value of 4.0 cm^−1^.

## 3. Results and Discussion

### 3.1. THz Spectral Characterization and Analysis of NF, MELA and Hydrated Co-Crystal

The action mechanism leading to THz characteristic absorption bands in various molecular systems is dominated by the excitation and resonant absorptions of both the intra-molecular and inter-molecular vibrational modes shown within different organic compounds from the perspective of dynamics. Therefore, the spectral features in the THz absorption spectra are usually considered to be dependent on intra- and/or inter-molecular behaviors including hydrogen bonding or other weak non-valence bonding interactions. In particular, it has potential applications in the pharmaceutical field for the analysis of crystalline compounds throughout the pharmaceutical development and research processes [22]. With the help of THz spectroscopic technology, it is possible to directly detect and differentiate different solid-state forms of APIs and their corresponding co-crystals, and to exploit structural change information for such specific pharmaceutical co-crystals whose crystal structures are stabilized by weak inter- and/or intra-molecular interactions between APIs and various CCFs [24,25].

**Figure 3:**
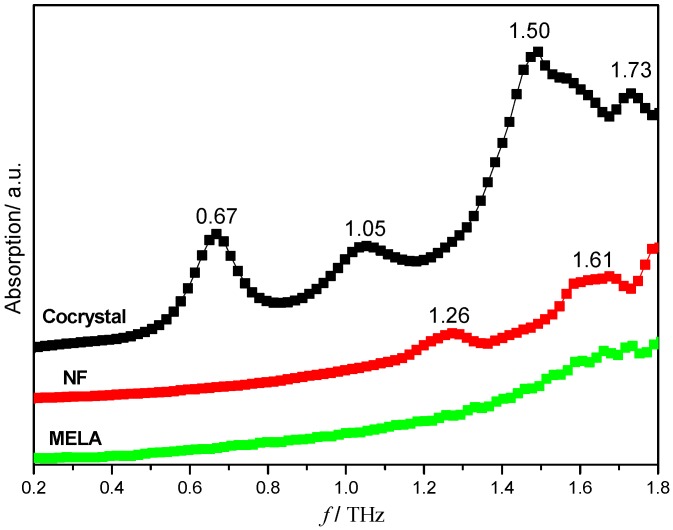
THz absorption spectra of NF, MELA and their hydrated co-crystal.

Figure 3 shows the THz absorption spectra of NF, MELA and their hydrated co-crystal, ranging from 0.2 to 1.8 THz. NF shows two characteristic peaks at the positions 1.26 and 1.61 THz, which is consistent with the anhydrous form of NF reported in our previous work [22], while MELA does not have any obvious absorption peaks during this spectral range. However, the corresponding NF–MELA-hydrated co-crystal has four peaks at 0.67, 1.05, 1.50 and 1.73 THz, respectively, and this is quite different to the two starting parent materials shown in the THz spectra. It could be primarily suggested that it is the effects of hydrogen bonding and the water molecule that lead to the structure change of the unit cells between the above two raw materials, along with the formation of the NF–MELA-hydrated co-crystal that results in the differences observed in the THz spectra between the NF–MELA-hydrated co-crystal and its parent compounds.

**Figure 4:**
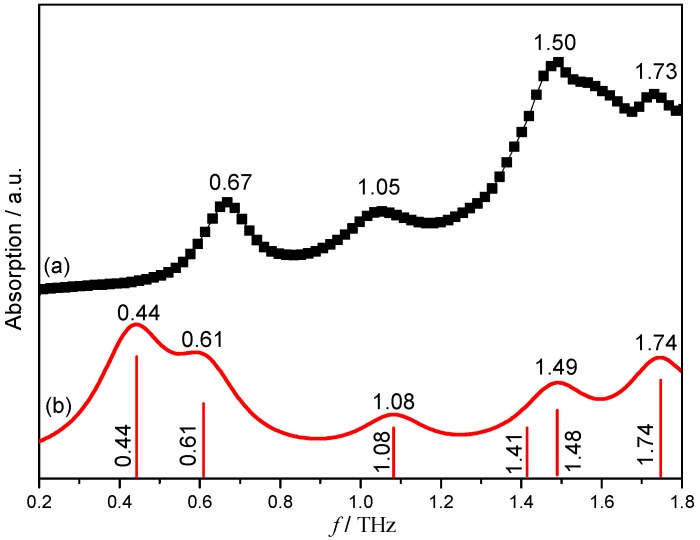
Experimental (**a**) and calculated (**b**) THz spectra of the hydrated co-crystal formed between NF and MELA.

Some theoretical simulations have been suggested as effective ways to bridge the observed low-frequency vibrational modes and the molecular structures and also the inter-molecular interactions shown within specific research materials. However, the explanation and understanding of the experimental vibrational modes shown in the THz spectra are still a fundamental scientific challenge [32,33,34,35]. The optimized geometry and the following vibrational mode simulations based on the hydrated co-crystal structure composed of NF, MELA and the water molecules with an equal molar ratio have been carried out by DFT calculation. The calculated and experimental results are relatively consistent with each other when compared with the simulated calculations and the experimental THz spectra of the NF–MELA-hydrated co-crystal, and this comparison is shown in Figure 4. The experimental band at 1.05 THz corresponds to the vibrational mode calculated at 1.08 THz, arising from the out-of-plane bending of the NF and MELA, as well as translation of the water molecule. The experimental peak at 1.50 THz could be attributed to the simulated mode at the position 1.49 THz, which arises from the out-of-plane bending of the NF and MELA, as well as translation of the water molecule. The observed peak at 1.73 THz is attributed to a combination of the in-plane bending of NF and out-of-plane bending of MELA.

The difference between the experimental and simulated THz spectral results is that, in the experimental spectra, there is only one peak at the position of 0.67 THz, while the theoretical calculation has two absorption peaks at 0.44 and 0.61 THz, respectively. The reason for such a spectral shift in the THz frequency region is the fact that the theoretical simulation is performed at an absolute zero degree temperature, while the experimental spectrum is obtained under ambient conditions. Similarly, the theoretical simulation is only a calculation of a single-molecule unit of the entire co-crystal, but the experimental THz absorption spectrum is a representation of the entire solid-state co-crystal network and may also cause some vibrational mode shift, and the exact spectral resolution of the experimental THz-TDS system could also lead to the appearance of such spectral difference. The complete description of the theoretical calculated vibrational modes of the NF–MELA-hydrated co-crystal is presented in Table 1.

### 3.2. Raman Spectral Characterization and Analysis of NF, MELA, Physical Mixture and Hydrated Co-Crystal

Raman vibrational spectra mainly represent the intra-molecular vibrations of the functional groups within pharmaceutical molecules, which have become a crucial and mainstream analytic technique to provide information about a specific structural fingerprint so that different materials can be exclusively characterized at the micro-molecular level and also identified under various conditions or physical/chemical processes. These vibrational modes can be affected by changes due to both molecular conformation and inter-molecular hydrogen bonding effects on the co-crystallization that is occurring.

**Figure 5:**
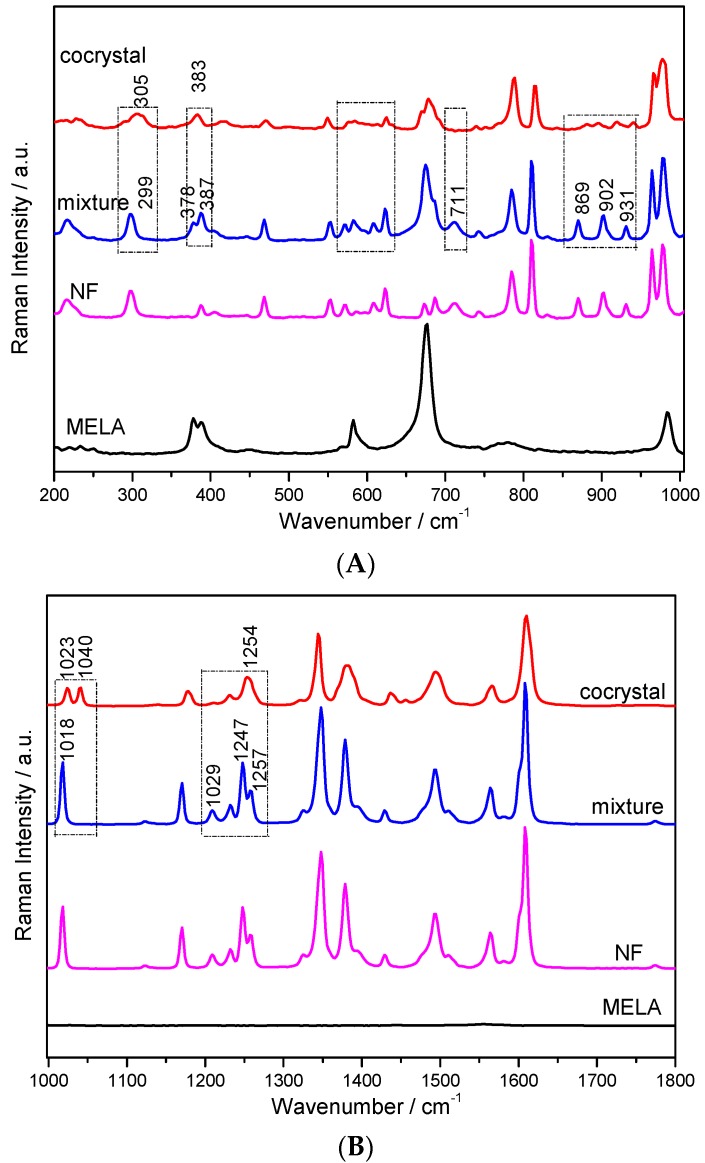
Raman spectra of NF, MELA, their physical mixture and the NF–MELA-hydrated co-crystal ranging from 200 to 1000 cm^−1^ (**A**) and 1000 to 1800 cm^−1^ (**B**).

The Raman vibrational spectra for the NF–MELA-hydrated co-crystal are distinctive compared with that for NF and MELA, shown in Figure 5. The intensities and/or vibrational modes shown in the Raman spectra are associated with the change in polarizability of specific chemical bonds within interested molecules. Due to the much more rigid and symmetrical structure of the MELA molecule, it shows relatively simple Raman bands (no evident vibrational bands in the 1000–1800 cm^−1^ region, shown in Figure 5B) compared to NF. It can be seen that the characteristic peaks of the physical mixture appear to be caused by a simple linear superposition of the individual spectra for the two parent components involved, NF and MELA, respectively. This means that there is no intermolecular interaction effect between NF and MELA without co-crystallization or another extraneous force. Meanwhile, the Raman spectrum of the NF–MELA-hydrated co-crystal form is different and has its own characteristic peaks compared to that of the physical mixture or the starting compounds. Several band shifts and new characteristic features could be observed in the whole spectral region, 200–1800 cm^−1^, as shown in Figure 5, in which the typical band locations are given next to the characteristic peaks. This Raman spectral evidence that the co-crystal is easily distinguishable compared with the involved starting materials is consistent with the results shown in the above THz spectra. It must be that the hydrogen bonding effect between the NF and MELA molecules leads to the structure change and appears as a new peak in the corresponding NF–MELA-hydrated co-crystal form at this position. In addition, except for the appearance or disappearance of the characteristic peaks, more peaks show some frequency-shift from their original positions along with the formation of the NF–MELA-hydrated co-crystal and a part of these typical Raman peaks is highlighted with dotted-line squares for easy observation in Figure 5.

**Figure 6:**
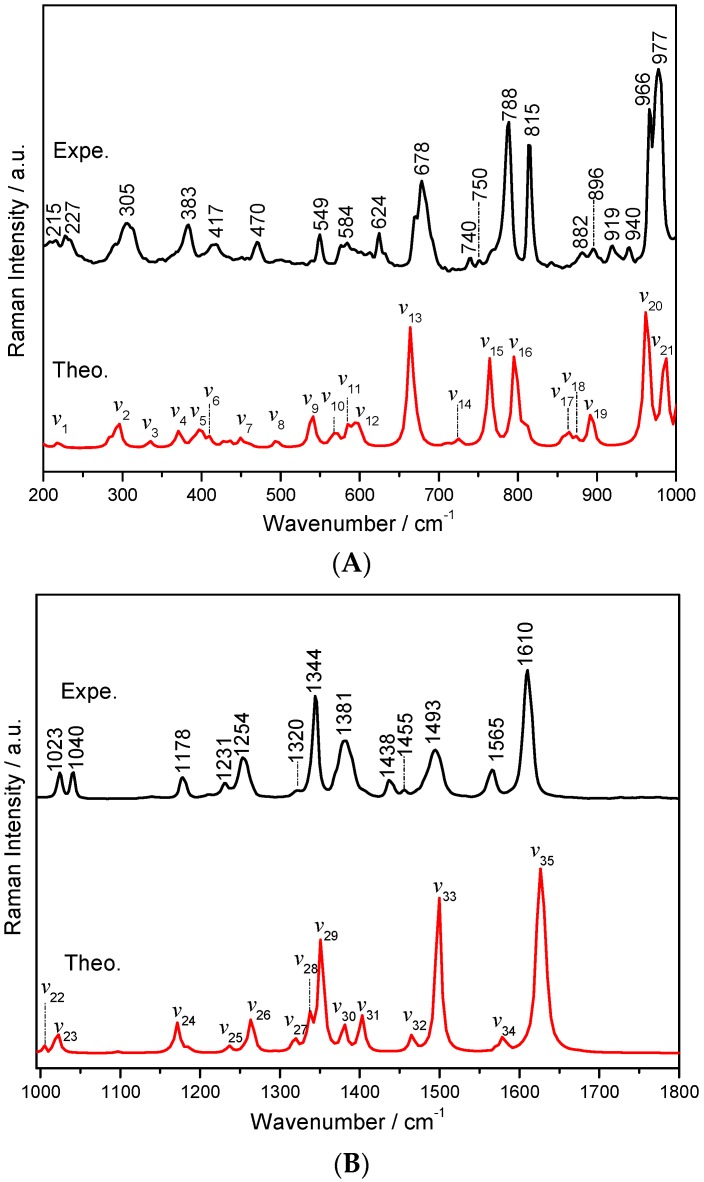
Comparison of the experimental (black line) and simulated (red line) Raman spectra of the NF–MELA-hydrated co-crystal ranging from 200 to 1000 cm^−1^ (**A**) and 1000 to 1800 cm^−1^ (**B**).

The theoretical DFT calculation of the Raman spectra revealed the vibrational modes of the hydrated co-crystal form between NF, MELA and the water molecule, and the comparison between the experimental and simulated results is shown in Figure 6. It could also be seen that both simulated and experimental spectra are relatively consistent with each other. Within the spectral region of 200~1000 cm^−1^, shown in Figure 6A, most of the vibrational modes in this region are from the deformation of rings and/or in-plane and out-of-plane bending of C–H and N–H bonds. The starting raw material, NF, has a band at 299 cm^−1^. It is a blue-shift to 305 cm^−1^ where it also becomes broader upon the formation of the hydrated co-crystal between NF and MELA. The intensity of some of the bands, such as 869, 902 and 931 cm^−1^, almost disappears, while the band at 788 cm^−1^ shows greater intensity and a broader width after the co-crystallization process. As for the spectral region from 1000 to 1800 cm^−1^, where most of the vibrational modes in this region are due to the stretching and bending vibrations of the functional groups, shown in Figure 6B, the intensities of the MELA vibrational modes are relatively weak compared with those of NF. In the Raman spectrum of the physical mixture, there is just one distinct peak at 1018 cm^−1^ which is only caused by the contribution from the NF molecule. However, a new peak appears at 1040 cm^−1^ in the hydrated co-crystal form and the peak in the physical mixture at 1018 cm^−1^ has been blue shifted to 1023 cm^−1^. The bands at 1029 and 1247 cm^−1^, shown in the physical mixture, disappear in that of the co-crystal, while the characteristic band at 1257 cm^−1^ is red-shifted to 1254 cm^−1^ upon the co-crystal formation. Based on the simulation results shown in Table 2, the band at 1344 cm^−1^ is mainly due to the stretching vibration of the nitro group in NF and the out-of/in-plane bending vibration of the N–H (the three amino groups) bond in MELA. Meanwhile, the band at 1610 cm^−1^ comes from the scissoring vibration of the amino groups within the MELA molecule combined with the out-of-plane bending vibration of the furan ring in the NF molecule. The peak at the position 1040 cm^−1^ in the experimental results is caused by the in-plane bending vibration of the C1–H17, C2–H18 and C6–H16 bonds which belong to the NF molecule. Regarding the position at 1023 cm^−1^, this peak originates from the deformation vibration of R3 and the in-plane bending vibration of the three amino groups belonging to MELA, and the out-of-plane bending vibration of the water molecule. It is known that the MELA molecule does not have any peaks at this position in the experimental spectra while a new peak appears at 1023 cm^−1^, and this is due to the contribution from the hydrogen bonding effect which is also in agreement with the THz spectral results mentioned above. The characteristic vibrational bands of the above NF–MELA-hydrated co-crystal are listed in Table 2 with complete vibrational mode assignments.

The Raman experimental spectra results indicated that the nitro group, the furan ring of NF, the water molecule and the amino group of MELA participate in hydrogen bonding formation via the inter-molecular interaction during its co-crystallization process. It also confirmed that the crystalline phase of the hydrated co-crystal is not simply a combination of the individual starting components but the totally different crystal phase due to the inter-molecular interaction effects, especially the hydrogen bonding interaction between the NF and MELA compounds.

The information obtained from the Raman vibrational spectra of the NF–MELA-hydrated co-crystal form is in agreement with that of the THz absorption spectra. It confirms that both Raman and THz spectroscopy could be promising alternatives to the mainstream traditional analytical tools, such as X-ray diffraction or thermal analysis, for further study of solid state co-crystal reactions in the pharmaceutical fields. Using THz and Raman spectroscopy to study the co-crystals formed between NF and MELA and then obtaining the spectra information could offer structural information about both the intra-molecular and inter-molecular interactions during the co-crystallization of active pharmaceutical ingredients. Combining the spectral results from these two vibrational spectroscopy techniques with simulated prediction could improve the analysis of both the co-crystal structural changes and the hydrogen bonding effects, and also help to obtain more comprehensive information for analyzing specific solid-state pharmaceuticals.

## 4. Conclusions

The vibrational spectra of solid-state NF, MELA and their hydrated co-crystal form were measured with THz-TDS and Raman vibrational spectroscopy. The experimental THz spectral results show that this NF–MELA-hydrated co-crystal has characteristic peaks at 0.67, 1.05, 1.50 and 1.73 THz, respectively, which is obviously different from the two corresponding raw starting materials. The Raman spectra also show some differences between the NF–MELA-hydrated co-crystal and the raw parent materials. DFT calculation was performed to simulate the optimized structure and the vibrational modes of the theoretical hydrated co-crystal form. The simulated calculations explain that both the Raman and THz theoretical spectra of the NF–MELA-hydrated co-crystal form are in a high degree of agreement with the experimental results. This study demonstrates the feasibility of taking advantage of vibrational spectroscopy for solid-state pharmaceutical applications and provides an experimental and theoretical benchmark for both the formation of co-crystals and the analysis of material structures and inter-molecular interactions (such as important hydrogen bonding effects) in the solid-state pharmaceutical research and development fields in the future.

## Figures and Tables

**Table 1:** Description of the vibrational modes of the NF–MELA-hydrated co-crystal in its THz spectrum.

Compound	Experimental Result (THz)	Theoretical Calculation (THz)	Vibrational Mode Assignment
NF–MELA-hydrated co-crystal	0.67	0.44	out-of-plane bending of NF and MELA; translation of H_2_O.
		0.61	out-of-plane bending of MELA; torsion of NF; translation of H_2_O.
	1.08	1.05	out-of-plane bending of NF and MELA; translation of H_2_O.
	1.50	1.41	out-of-plane bending of NF and MELA; translation of H_2_O.
		1.49	out-of-plane bending of NF and MELA; translation of H_2_O.
	1.73	1.74	in-plane bending of NF; out-of-plane bending of MELA.

**Table 2:** Vibrational mode assignment for the Raman characteristic peaks of the NF–MELA-hydrated co-crystal.

Mode	Theoretical/cm^−1^	Experimental/cm^−1^	Vibrational Mode Assignment
ν_1_	219	227	ρ(–NO_2_, R1)
ν_2_	294	305	τ(H_2_O), ρ(R2), ω(N33H36H37)
ν_3_	345	—	ρ(N33H36H37, N34H40H41, N35H38H39)
ν_4_	373	383	Def R1, ω(C6H16), ρ(H_2_O, N33H36H37, N34H40H41, N35H38H39)
ν_5_	399	417	ω(N34H40H41)
ν_6_	410		ω(N35H38H39)
ν_7_	450	470	ω(O25H24, N33H36), Def R1
ν_8_	495	—	τ(N34H40H41)
ν_9_	541	549	ρ(–NO2, R1, R2, C9H2122), ω(N12H23)
ν_10_	570	584	Def R3
ν_11_	585	—	Def R2, R1
ν_12_	597	624	Def R2, ρ(C11O19, N12H23), ω(C9H21H22)
ν_13_	664	678	Def R3, ω(O25H26)
ν_14_	725	740	Def R1, ρ(–NO2), ω(N35H38, C1H17)
ν_15_	764	788	Def R1, R2, ρ(C6H16, N12H23)
ν_16_	795	815	δ(-NO_2_), Def R1
ν_17_	864	882	Def R1, R2, ρ(N12H23), ω(C9H21H22)
ν_18_	873	896	ω(C1H17, C2H18, C6H16)
ν_19_	893	919	ω(C6H16, C1H17, C2H18)
ν_20_	962	966	Def R3, R1
ν_21_	986	977	Def R1, ρ(C1H17, C2H18, C6H16)
ν_22_	1008	1023	Def R3, ω(O25H26), ρ(N33H36H37, N34H40H41, N35H38H39)
ν_23_	1020	1040	ρ(C1H17, C2H18, C6H16)
ν_24_	1172	1178	ρ(C1H17, C2H18, C6H16), Def R1
ν_25_	1237	1231	Def R1, ω(C9H21H22), θ(N7N8)
ν_26_	1265	1254	Def R1, ρ(C1H17, C2H18, C6H16), θ(–NO2)
ν_27_	1318	1320	ρ(N12H23, C6H16), ω(C9H21H22)
ν_28_	1338	1344	ρ(N12H23, C1H17, C2H18, C6H16)
ν_29_	1352		θ(–NO_2_), Def R1, R2, ρ(N12H23, C6H16), ω(C9H21H22)
ν_30_	1380	1381	θ(–NO_2_), Def R1, R2, ρ(N12H23, C6H16, C1H17, C2H18), ω(C9H21H22)
ν_31_	1403		Def R1, ρ(C6H16, C1H17, C2H18)
ν_32_	1466	1455	ρ(C9H21H22)
ν_33_	1499	1493	Def R1, R3, ρ(C1H17, C2H18), δ( N33H36H37, N34H40H41, N35H38H39)
ν_34_	1579	1565	δ(N33H36H37, N34H40H41, N35H38H39), Def R1, R3, θ(–NO_2_)
ν_35_	1627	1610	ω(C3C6N7), ρ(C6H16), δ(N33H36H37, N34H40H41, N35H38H39)

θ—stretching, ρ—in-plane bending vibration, ω—out-of-plane bending vibration, τ—torsion, δ—scissor, Def-deformation, R1, R2—five-member ring in NF shown in Figure 2, R3—ring in MELA shown in Figure 2.
